# Photoperiodic plasticity in circadian clock neurons in insects

**DOI:** 10.3389/fphys.2013.00069

**Published:** 2013-08-23

**Authors:** Sakiko Shiga

**Affiliations:** Department of Biology and Geosciences, Graduate School of Science, Osaka City UniversityOsaka, Japan

**Keywords:** photoperiodism, circadian clock neurons, plasticity, Per, s-LNv, pigment-dispersing factor

## Abstract

Since Bünning's observation of circadian rhythms and photoperiodism in the runner bean *Phaseolus multiflorus* in 1936, many studies have shown that photoperiodism is based on the circadian clock system. In insects, involvement of circadian clock genes or neurons has been recently shown in the photoperiodic control of developmental arrests, diapause. Photoperiod sets peaks of *period* (*per*) or *timeless* (*tim*) mRNA abundance at lights-off in *Sarcophaga crassipalpis*, *Chymomyza costata* and *Protophormia terraenovae*. Abundance of *per* and *Clock* mRNA changes by photoperiod in *Pyrrhocoris apterus*. Subcellular Per distribution in circadian clock neurons changes with photoperiod in *P. terraenovae*. Although photoperiodism is not known in *Leucophaea maderae*, under longer day length, more stomata and longer commissural fibers of circadian clock neurons have been found. These plastic changes in the circadian clock neurons could be an important constituent for photoperiodic clock mechanisms to integrate repetitive photoperiodic information and produce different outputs based on day length.

## Introduction

Photoperiod is an important cue for organisms to obtain information on calendar time from the environment. Plants and animals respond to the regular changes in day length to coordinate their development and physiology to these seasonal changes. This is called photoperiodism. This review focuses on plasticity or changes in gene expression and neural morphology that might be involved in photoperiodic mechanisms in insects. The physiological mechanisms underlying photoperiodism comprise three components: photoperiodic photoreceptors, photoperiodic clocks, and endocrine systems. Photoperiodic information is processed by neural elements responsible for the photoperiodic clock. The outputs from the photoperiodic clock affect the endocrine systems in which the release of humoral factors controls developmental programs (Saunders, 2002). The photoperiodic clock has been considered to consist of a two-step process: one step being the time-measurement system, which measures the day or night portion of a day and the other is the photoperiodic counter. In the latter step, photoperiodic information is successively received during the sensitive period and it then accumulates to an internal threshold. Once this threshold is passed, the endocrine events controlling development are triggered. It is quite interesting how the brain integrates repetitive photoperiodic information as well as whether any neural plasticity is involved in the photoperiodic clock. However, very few studies have addressed these issues.

Bünning (1936) had first proposed that an endogenous circadian oscillator is involved in the time-measurement system for photoperiodic responses. His original idea was based on the observations of leaf movements in the runner bean *Phaseolus multiflorus*. He found two distinct phases comprising a circadian cycle: one is called “photophil,” a light-requiring phase (upward movement of the leaf), and the other, “scotophil,” a darkness-requiring phase (downward movement of the leaf). *P. multiflorus* exhibits photoperiodism and its flowering is promoted under short-day conditions. Bünning found that longer period of exposure to light during scotophil phase in *P. multiflorus* delayed the flowering time compared to shorter period of exposure to light (Bünning, 1936). He reached the idea that photoperiod is measured by referring to the phases of the circadian clock that are entrained to environmental light and dark cycles. Since then, many pieces of experimental evidence have supported the circadian clock-based time-measurement, but only recently the involvement of circadian clock genes or neurons has been shown in case of insects.

In the temperate region insects grow and reproduce in favorable seasons whereas they enter diapause, developmental arrest, in unfavorable seasons. Diapause is controlled by photoperiod in many insects. In-depth molecular studies of the circadian clock mechanism have been successfully undertaken in *Drosophila melanogaster* (Peschel and Helfrich-Förster, 2011), but the temperature is the main determinant of dormancy, and photoperiod has small effects on it (Emerson et al., 2009; Hahn and Denlinger, 2011). Therefore, the involvement of circadian clock mechanisms in photoperiodism has been analyzed in different species. Some clear results were obtained showing the necessity of circadian clock genes in the photoperiodic control of diapause by using mutant analysis or RNA interference technique. A circadian clock gene *timeless* (*tim*) has been shown to be an important gene for the photoperiodic response of a drosophilid fly *Chymomyza costata*, *period* (*per*) for the cricket *Modicogryllus siamensis*, and both *per* and *Cycle* (*Cyc*) for the bean bug *Riptortus pedestris* (Pavelka et al., 2003; Sakamoto et al., 2009; Ikeno et al., 2010). Ablation experiments have suggested that circadian clock neurons driving locomotor activity rhythms are prerequisite to photoperiodism in the blow fly *Protophormia terraenovae* (Shiga and Numata, 2009). However, it still has to be unveiled how these circadian clock genes or neurons play roles in photoperiodism.

It is unknown how photoperiodic information is encoded in the neural system. Photoperiod is composed of a fixed period (*t* h) of light and 24-*t* h of darkness. Discrimination of light from darkness can be simple, but measuring *t* or 24-*t* h and discriminating long days from short days are more complex processes. Encoding photoperiodic information requires a time-coding system. This can be handled by two ways: one refers to the circadian clock phases (described by Bünning) and the other uses a non-oscillating hourglass, which is set in motion in each cycle at light-off and measure night length by referring to critical night length (Putterill et al., 2010). It is quite possible that coding information in a rather long time period needs neural plasticity. As no idea is so far available for hourglass mechanisms by neurons, here I discuss aspects of plasticity in gene expression, subcellular clock protein location and neural morphology that are observed in circadian clock neurons under different photoperiods.

## Photoperiodic changes in clock gene expression

Studies on the molecular machinery underlying the circadian clock in *D. melanogaster* show that many clock genes and proteins interacting in at least three interdependent feedback loops are involved in rhythm generation (Peschel and Helfrich-Förster, 2011). Circadian fluctuations in the abundance of *per* and *tim* mRNA and their proteins are an important element in the feedback loops. One important characteristic of the circadian oscillator is entrainment to the environmental cycles. The molecular basis of entrainment mechanisms to light-dark cycles (photoperiodic cycles) has been understood partially. During photoperiodic cycles, Cryptochrome (Cry) is activated by “lights-on” leading to Timeless (Tim) degradation, and because of the lack of Tim, Per is vulnerable to phosphorylation and subsequently degraded, thus starting a new cycle (Lin et al., 2001). The Cry signal is terminated by “lights-off” leading to nighttime accumulation of Per and Tim (Qiu and Hardin, 1996). Thus, Cry photoreception in the clock neurons is a key feature of photoperiodic entrainment. Considering their characteristics under different photoperiods, the temporal accumulation patterns of the circadian clock mRNA and proteins can be expected to undergo changes. In fact, the mRNA of *per* and *tim* of the head sample reach their peaks in abundance 4 h after “light-off” during photoperiods of 16-h light and 8-h darkness (LD, 16:8), LD 12:12, and LD 8:16, and oscillation uses “lights-off” as a phase reference point (Qiu and Hardin, 1996). Setting peaks of *per* or *tim* mRNA levels at “lights-off” have also been observed in the head of the flesh fly *Sarcophaga crassipalpis*, the whole central nervous system of *C. costata*, and the brain of *P. terraenovae* (Goto and Denlinger, 2002; Stehlík et al., 2008; Muguruma et al., 2010). Other types of responses of circadian clock gene expression to photoperiods have also been observed. In the head of the linden bug *Pyrrhocoris apterus*, clear oscillation has not been observed, but *per* mRNA abundance was 10-fold higher and *Clock* mRNA was 2-fold higher under long-day conditions than under short-day conditions (Syrová et al., 2003). The circadian clock gene expression responds to photoperiods, although the responses vary among species.

## Photoperiodic changes in the subcellular location of circadian clock proteins

In the current model of the circadian clock system in *D. melanogaster*, the very center of the clock mechanism is characterized by the activation of *per* and *tim*. The evening-time increase in mRNA leads to the accumulation of Per and Tim in the cytoplasm. Tim and Per enter the nucleus alone or as a heterodimer allowing Per-associated double-time (Dbt) kinase to co-enter the nucleus. This transport is mediated via Per phosphorylation. Inside the nucleus, the Per-Dbt-Tim complex accumulates and binds to Clock (Clk)/Cycle (Cyc) dimers via Per-Clk interaction, causing the hyperphosphorylation of Clk to prevent Clk/Cyc dimers from binding to E-Box sequences in the promoter region of many circadian clock-regulated genes, including *per* and *tim*. This inhibits the transcription of *tim* and *per* toward “lights-on.” Per's inhibition of its own transcription generates a negative feedback loop. Cyclic expression of Per and Tim protein and mRNA have been observed previously (Peschel and Helfrich-Förster, 2011).

Using transgenic lines or immunocytochemistry, the subcellular distribution of the circadian clock proteins has been examined. In *D. melanogaster*, about 150 neurons express circadian clock genes in the brain. These neurons are classified into seven groups and designated according to their anatomical locations. By using markers recognizing molecules involved in circadian clock systems, each group of clock neurons was further divided into several subgroups (Kaneko and Hall, 2000; Peschel and Helfrich-Förster, 2011). Among them, the small type of ventral lateral neurons (s-LN_v_s) was considered the main circadian oscillator for behavioral rhythm under constant darkness. The effects of day length on the temporal profiles of Per and Tim accumulation in the nuclei or cytoplasm have been studied in s-LN_v_s. The temporal patterns of the subcellular distribution of clock proteins change according to the photoperiod: both Tim and Per were almost absent in the cell shortly after “lights-off,” and at “lights-on” protein accumulation was obvious in the nuclei, especially in Per during both long-day and short-day conditions (Shafer et al., 2004). This means that nuclear localization timing is affected by photoperiod in *D. melanogaster*. In addition, in *P. terraenovae*, a similar distribution of Per-immunoreactive cells to *D. melanogaster* has been revealed (Shiga and Numata, 2009). The subcellular distribution of Per-immunoreactivity was compared between the long-day and short-day conditions in four types of circadian clock cells, namely, s-LN_v_s, large type of ventral lateral neurons (l-LN_v_s), dorsal lateral neurons (LN_d_s), and medial dorsal neurons (DN_m_s) (Muguruma et al., 2010). In all cell types, Per-immunoreactivity in the nucleus was the highest 12 h after “lights-off” and lowest 12 h after “lights-on” irrespective of photoperiod. This results in a clear differences in Per distribution at “lights-on” between the photoperiod: under short days, Per is mainly localized in the nucleus, whereas Per-immuno-positive nuclei were less in number under long-day conditions at “lights-on.” Per nuclear translocation seems to entrain to photoperiod. The temporal patterns of Per staining under short-day conditions slightly differed among the cell types of *P. terraenovae*. In l-LN_v_s and s-LN_v_s, Per remains in the nucleus for a longer period of time during photophase than in LN_d_s and DN_m_s. It is known that in *D. melanogaster*, Per is stabilized by Tim, but without protection by Tim during photophase Per is phosphorylated and degraded. The degradation process of Per might be slow in the s-LN_v_s and l-LN_v_s, compared with LN_d_s and DN_m_s in *P. terraenovae*.

In *D. melanogaster*, two circadian oscillators, namely, morning and evening oscillators, have been functionally proposed to drive locomotor rhythms. Stoleru et al. (2007) suggested differential circadian photo-entrainment features present between the “evening cells” of LN_d_s, DN1s, and DN2s, and “morning cells” of sLN_v_s. These features with inter-oscillator communication may underlie circadian adjustment to the changes in photoperiod. GFP expression driven by clock gene promoters has very well-revealed the fiber distribution of circadian clock neurons (Helfrich-Förster, 2003). The clock neurons form a network with their fine terminals in dorsal protocerebrum, which houses fibers from the neurosecretory cells in the brain. This circadian neural network must encode day-length information, and it may submit the output signals to the dorsal protocerebrum, where neurosecretory cells control diapause or reproduction (Shiga and Numata, 2000).

## Circadian and photoperiodic changes in fiber distribution in clock neurons

Fiber distribution of the circadian clock neurons also shows daily plasticity in *D. melanogaster* (Fernández et al., 2008). By using a membrane-bound version of GFP under the *pigment-dispersing factor* (*pdf*)-specific promoter, the morphological plasticity of the terminal fibers of s-LN_v_s was examined. The s-LN_v_s have somata at the ventral and anterior bases of the medulla in the optic lobe, and extend their axonal fibers posterior-dorsally to the protocerebrum. In the dorsal protocerebrum s-LN_v_s make trajectory shifts and open varicose fibers two-dimensionally by the somata of DN1s and DN2s. As the s-LN_v_ branches in the dorsal protocerebrum are labeled by a presynaptic marker, these branches are considered terminal sites. The rhythmic alterations in the structure were found to contain higher complexity in fiber distribution during the day, and significantly lower complexity during the night. These oscillations continue under both light-dark cycles of 24 h and constant dark conditions. These structural changes disappear in the clock-less mutants of *D. melanogaster*. These morphological changes could be a mechanism for transmitting clock information in a time-dependent manner, and may indicate a change in the terminal fibers of s-LN_v_s connecting to different targets at different times during the day (Fernández et al., 2008).

PDF-immunoreactive neurons in the cockroach *Leucophaea maderae* show morphological plasticity when reared under different photoperiods (Wei and Stengl, 2011). In the cockroach, the circadian clock controlling the locomotor activity rhythms is located at the accessory medulla with associated PDF-immunoreactive neurons. According to cell size and location, PDF-immunoreactive neurons are classified into five types. One type called medium-sized aPDFMes obviously responds to changes in day length. With the increase in day length, the amount of PDF-immunoreactive somata has increased and longer commissural fibers have been found. Because visual stimulation or monocular deprivation during development causes changes in optic lobe volume, this difference could be simply caused by difference in visual experience (Barth et al., 1997). However, considering the fact that photoperiod seems to differentially affect PDF-ir neurons, this photoperiodic change in medium-sized aPDFMes suggests some role in photoperiodic adjustment in the clock mechanism. Because *L. maderae* is a tropical species as is *D. melanogaster*, this does not show phenotypic plasticity like diapause induction by photoperiod. It would be interesting to investigate morphological plasticity in the circadian clock neurons in insects showing clear photoperiodism.

During the examination of physiological mechanisms underlying photoperiodism, molecular and neural activities are easily taken into account. However, dynamic changes of neural morphology may be one approach to investigate neural coding of photoperiodic information. In the Japanese quail *Coturnix japonica*, which shows clear photoperiodism controlling gonadal maturation, morphological changes are known to occur in the terminal sites of the gonadotropin-releasing hormonal neurons under different photoperiods (Yamamura et al., 2004). Immuno-electron microscopy revealed that gonadotropin-releasing hormonal nerve terminals are in close proximity to the pericapillary space for secreting neurohormone into portal blood under long days, but that their terminals are encased by glial cells under short-day conditions. In future, it might prove interesting to examine the differences in morphological plasticity in the circadian clock neurons between long-day and short-day conditions in insects showing clear photoperiodism.

## Conclusion

Photoperiodic changes have been reported in the expression patterns of the circadian clock genes, in the subcellular distribution of clock proteins, and in fiber distribution or the number of clock neurons. These plastic changes in the circadian clock neurons could tell photoperiodic information to some unknown neuronal networks, where day counting must occur up to a certain threshold. When the number of days passes the threshold, neurosecretory systems are excited or suppressed to control development and diapause. Because circadian output fibers are intermingled, and neurosecretory cells in the pars intercerebralis and pars lateralis are located, the dorsal protocerebrum could furnish important neural networks for photoperiodic clock system.

### Conflict of interest statement

The author declares that the research was conducted in the absence of any commercial or financial relationships that could be construed as a potential conflict of interest.
